# Pulse pressure is associated with cognitive performance in Japanese non-demented population: a cross-sectional study

**DOI:** 10.1186/s12883-022-02666-6

**Published:** 2022-04-11

**Authors:** Ryo Mizuhara, Shingo Mitaki, Masahiro Takamura, Satoshi Abe, Keiichi Onoda, Shuhei Yamaguchi, Atsushi Nagai

**Affiliations:** 1 Department of Neurology, National Hospital Organization Maizuru Medical Center, 2410 Yukinaga, Maizuru, Kyoto 625-8502 Japan; 2 Department of Neurology, Shimane University School of Medicine, 89-1 Enya-cho, Izumo, Shimane 693-8501 Japan; 3 Faculty of Psychology, Otemon Gakuin University, 2-1-15 Nishiai, Ibaraki, Osaka 567-8502 Japan; 4 Shimane Prefectural Central Hospital, 4-1-1 Himebara, Izumo, Shimane 693-8555 Japan

**Keywords:** Pulse pressure, Cognitive functions, Propensity-matching, Mediation analyses

## Abstract

**Background:**

Growing evidence suggests that vascular risk factors, especially hypertension, relate not only to cardiovascular disease but also to cognitive impairment. However, the impact of pulse pressure on cognitive function remains controversial. In this study, we evaluated the associations between pulse pressure and cognitive function in a Japanese health examination cohort using propensity matching analysis.

**Methods:**

We examined 2,546 individuals with a mean age of 60.8 ± 10.3 years who voluntarily participated in health examination. Clinical variables included pulse pressure, and brain magnetic resonance imaging (MRI). We divided the participants into the high and low pulse pressure groups with a pre-defined cut-off value of 65 mmHg and evaluated their physical examination data, cognitive functions including Okabe’s test, Kohs’ test, and silent brain lesions using propensity matching. To clarify whether pulse pressure and blood pressure have different implications for cognitive function, a mediating analysis was also conducted.

**Results:**

From the 2,546 subjects, 439 (17.2%) were in the high PP group. The propensity matching algorithm produced 433 pairs of patients with similar propensities. Higher pulse pressure corresponded to lower Okabe and Kohs’ scores (44.3 ± 7.1 vs 42.7 ± 7.5; *p* = 0.002, 97.9 ± 18.0 vs 95.0 ± 18.1 *p* = 0.019, respectively). The relationship between pulse pressure and cognitive impairment was not significantly mediated by systolic blood pressure. We observed no significant associations between silent brain lesions and pulse pressure.

**Conclusion:**

High pulse pressure was associated with lower cognitive performance without systolic blood pressure mediation in Japanese subjects without dementia.

**Supplementary Information:**

The online version contains supplementary material available at 10.1186/s12883-022-02666-6.

## Background

Pulse pressure (PP) is calculated as the difference between systolic (SBP) and diastolic blood pressure (DBP) and is considered a measure of arterial stiffness. Recently, high pulse pressure was linked to increased risk of cardiovascular disease [1]. Moreover, because changes in PP can occur in accordance with both SBP and DBP fluctuations, PP might be more useful than blood pressure (BP) for predicting cardiovascular risks [2].

Vascular risk factors, especially hypertension, are known to be related not only to cardiovascular disease but also to cognitive impairment [3]. Since hypertension is a major risk factor for cerebrovascular disease, it may also be associated with vascular dementia. Moreover, recent studies suggests that vascular risk factors influence the clinical course and pathophysiology of Alzheimer’s disease [4] and the association between BP and cognitive impairment has been observed in several epidemiological studies [5]. Given that PP is a better clinical indicator of functional arterial changes than BP itself, it might be related to the pathogenesis of cognitive impairment. However, there is conflicting evidence about the association of cognitive performance with PP. Several studies have suggested that high PP is associated with worse cognitive performance [6–9]. However, opposite results have also been demonstrated in some studies [10, 11]. There are indications that an age effect could partly explain these conflicting findings.

In this study, we evaluated the associations between PP and cognitive function in a well-characterized Japanese health examination cohort using two complementary statistical approaches: propensity matching and multivariate regression.

## Methods

### Study population

We studied a total of 2,546 individuals (1,386 men and 1160 women) with a mean age of 60.8 ± 10.3 years (range 27–95). All participants voluntarily underwent the brain health check-up at the Shimane Health Science Center between April 2004 and July 2015. The assessment included medical history, neurological examination by an experienced neurologist, BP measurement, neuropsychological testing, and magnetic resonance imaging (MRI) scans of the head. The criteria for subject exclusion were as follows: any history of neurological or psychiatric conditions, such as cerebrovascular diseases including transient ischemic attack, dementia, depression, or other psychiatric diseases, and missing data. All individuals provided informed consent to participate in this study, which was approved by the institutional ethics committee.

### Physical examination

BP was measured twice in the brachial artery of seated participants using an automatic electronic device after 5 min of rest. The mean of the two measurements was used. Hypertension was defined as having a SBP > 140 mmHg, a DBP > 90 mmHg, or a history of hypertension with antihypertensive therapy based on the Guidelines from the 2019 Japanese society of hypertension [12]. Diabetes mellitus was defined by fasting glucose level exceeding 126 mg/dl, random glucose level exceeding 200 mg/dl, an HbA1c level exceeding 6.5%, and/or a medical or self-reported history of diabetes or treatment with oral antidiabetic drugs or insulin. Dyslipidemia was defined by serum triglyceride level exceeding 150 mg/dl, high-density lipoprotein cholesterol level below 40 mg/dl, or a medical history of dyslipidemia.

### Brain imaging

Brain infarction was defined as a focal hyperintense lesion ≥ 3 mm in diameter on T2-weighted images. Fluid-attenuated inversion recovery images were used to differentiate infarcts from enlarged perivascular spaces. Cerebral microbleeds (CMBs) were defined as homogenous round foci of signal loss on gradient-echo T2*-weighted images that were 2–10 mm in diameter. Periventricular hyperintensities (PVHs) and white matter hyperintensities (WMHs) were evaluated based on their distinct subcortical distributions on fluid-attenuated inversion recovery images. PVH and subcortical white matter hyperintensity (SWML) were evaluated separately based on their distinct subcortical distributions on the fluid-attenuated inversion recovery image, because PVH was observed adjacent to the ventricles and SWML was observed separately from the ventricles. PVH was graded on a scale of 0 – 4, as described previously [13]. SWML was graded on a scale of 0 – 3 according to the Fazekas grading scheme [14]. For statistical purposes, PVH and SWML grades were dichotomized; we defined PVH grades 0–2 as ‘PVH–’ and grades 3–4 as ‘PVH + ’; similarly, SWML grades 0–1 were defined as ‘SWML–’, and grades 2–3 were termed ‘SWML + ’. CMBs were identified as 2–10 mm diameter rounded hypointense lesions on T2*-weighted images. All MRI findings were evaluated separately by an experienced neurologist and a radiologist who were blinded to the patient profiles. When their opinions were inconsistent, a second neurologist was consulted. An interrater study for evaluating MRI lesions was performed blindly by two independent raters.

### Cognitive function evaluation

General cognitive function was assessed using Okabe’s Intelligence Scale (Okabe’s test) [15], which is a shortened and modified Wechsler Adult Intelligence Scale-Revised for the Japanese aged population and includes orientation, semantic memory, calculation, forward and backward digit span, and paired association memory. The test scores a total of 60 points, and its reliability has been previously validated [16]. There was a significant correlation between Okabe’s test and the Mini Mental State Examination (MMSE) [15, 16]. The Kohs’ block design test (Kohs’ test) is a popular bedside screening test for constructional function and cognitive function. The subjects were shown cards with a variety of colored designs and were asked to reproduce them using a set of colored blocks, yielding an intelligence quotient [17]. This test assessed the visuospatial ability in addition to executive function. Frontal function was estimated using the frontal assessment battery (FAB) [18]. Affective functions were evaluated using the Self-rating Depression Scale (SDS) [19] and the Japanese version of the Apathy Scale [20].

### Statistics

In order to examine the effect of PP on several physical and functional assessments, we applied two statistical approaches: 1) a comparison between high and low PP groups with propensity score matching, and 2) a multiple regression analysis. In the former approach, we divided all subjects into high and low PP groups with a pre-defined cut-off value of 65 mmHg [21]. To avoid possible confounding effects caused by grouping with PP values, we used propensity score matching in this study. To obtain the propensity score, we assigned these two groups to the explanatory variable and performed logistic regression analysis after correcting for the following covariates suggested to influence pulse pressure: age, sex, and medical history of hypertension, diabetes mellitus, and dyslipidemia. Propensity score matching was performed using the following algorithm: 1:1 ratio nearest-neighbor match with ± 0.01 caliper and no replacement. The balance diagnostics were tested using standardized difference (*d*), calculated as following [22]. For a continuous covariate,

$$d=\frac{{\overline{x} }_{highPP }- {\overline{x} }_{lowPP }}{\sqrt{\frac{{var}_{highPP}+ {var}_{lowPP}}{2}}}$$

where ${\overline{x} }_{group}$ and ${var}_{group}$ denote the mean and sample variance of the covariate in each group, respectively. For categorical or dichotomous covariates,

$$d=\frac{\left({\widehat{p}}_{highPP}- {\widehat{p}}_{lowPP}\right)}{\sqrt{\frac{{\widehat{p}}_{highPP}\left(1-{\widehat{p}}_{highPP}\right)+ {\widehat{p}}_{lowPP}\left(1-{\widehat{p}}_{lowPP}\right)}{2}}}$$

where ${\widehat{p}}_{group}$ denote the mean prevalence of the dichotomous variable in each group.

Physical examination results, silent brain lesions, and cognitive functions were compared between the high and low PP groups, before propensity matching, using a two-sided Student’s t-test for continuous variables and χ^2^ analyses for categorical variables. After matching, two-sided paired t-test and McNemar test were used for the group comparison, respectively. To evaluate whether the association between PP and cognitive function was affected by SBP, mediation analysis was performed. In the mediation analysis, the significance of the effect was tested using Sobel’s delta method. None of the control variables were included in the mediation model.

As an additional analytical approach, we conducted multiple regression analyses for cognitive function variables. In the initial propensity score matching approach, the two PP groups were generated by dichotomizing a continuous variable with a cut-off value. Although the applied cut-off value has been used in prior studies [21], this procedure is criticized because it can induce underpower [23]. In this regression analyses, PP value was used as a continuous explanatory variable and all study samples were subjected to the analysis. In the multiple regression model, PP, SBP, age, sex, medical history of hypertension, diabetes mellitus, and dyslipidemia were included as explanatory variables. SBP was included to determine the variable related to the dependent variable. Dependent variables were selected based on the result of the propensity matching approach.

All statistical analyses were performed using IBM SPSS Statistics ver. 22 (SPSS, Inc.). Differences were considered significant at *p* < 0.05.

## Results

### Physical examination data

Population statistics of the PP groups were characterized before propensity matching as presented in Table 1. Among 2,546 participants, 439 (17.2%) were in the high PP group. Individuals in the high PP group were older (*p* < 0.001), and were more often females, compared with the low PP group (*p* = 0.003). Subjects with higher PP had hypertension (*p* < 0.001) and diabetes (*p* < 0.001) significantly more often. However, there was no significant difference in the prevalence of dyslipidemia between the groups. The propensity matching algorithm produced 433 pairs of patients with similar propensities. The standardized difference scores of the controlled variables were small enough (d < 0.1). In the propensity-matched dataset, the SBP and DBP were higher in persons with high PP than in those with low PP (*p* < 0.05).

**Table 1:** Baseline characteristics before and after propensity matching

Characteristics	Before matching			After matching		
	Pulse pressure (mmHg)			Pulse pressure (mmHg)		
	Lower (< 65)	Higher (≧65)	*P* value	Lower (< 65)	Higher (≧65)	Balance statistics
No. of subjects	2017	439		433	433	
Age (year)	59.8 (10.4)	65.4 (8.5)	< 0.001	64.9 (8.6)	65.4 (8.4)	0.030
Female (%)	46.6	51.9	0.003	50.3	51.5	-0.023
Hypertension (%)	32	59.2	< 0.001	60.2	58.9	-0.028
Diabetes (%)	9	14.1	< 0.001	13.4	13.6	0.007
Hyperlipidemia (%)	48.3	50.8	0.087	52	50.6	-0.028

### Brain imaging results

The prevalence of silent brain infarct (SBI), PVH, SWML, and CMBs is shown for each group after propensity matching in Table 2. For information, the metrics of before matching are presented in Suppremental table. Before matching, subjects with higher PP had more SBIs (*p* < 0.001) and worse PVH (*p* < 0.001) and SWML (*p* < 0.001) scores (Supplemental Table). In the propensity-matched sample, there were no significant differences in the occurrence of silent lesions between the two groups.

**Table 2:** Physical, brain, and cognitive assessment in participants with lower and higher pulse pressure after matching

	Pulse pressure (mmHg)		*P* value^a^
	Lower (< 65)	Higher (≧65)	
Systolic BP (mmHg)	127.5(14.2)	149.9(14.1)	< 0.001
Diastolic BP (mmHg)	73.7(11.4)	75.9(11.5)	0.005
PP (mmHg)	53.9(7.6)	74.0(7.3)	< 0.001
SBI	35 (8.1)	45 (10.4)	0.282
PVH grade≧3	49 (11.3)	49 (11.3)	1.000
SWML	127 (29.3)	143 (33.0)	0.217
CMBs	44 (9.9)	32 (7.4)	0.182
Okabe’s test (shortened version of WAIS-R)	44.3 (7.1)	42.7 (7.5)	0.001
Information	16.2 (2.8)	15.9 (2.7)	0.118
Mental control	11.9 (3.8)	11.1 (4.1)	0.001
Digit span	9.0 (1.4)	8.8 (1.5)	0.011
Assoc. learning	7.1 (3.0)	7.0 (3.1)	0.492
Kohs’ test	97.9 (18.0)	95.0 (18.1)	0.010
FAB	15.8 (1.5)	15.7 (1.5)	0.165
SDS	34.4 (7.9)	33.8 (7.7)	0.229
Apathy scale	10.9 (5.4)	11.2 (5.7)	0.453

*Abbreviation: BP* Blood Pressure, *SBI* Silent Brain Infarction, *PVH* Periventricular Hyperintensity, *SWML* Subcortical White Matter Hyperintensity, *CMBs* Cerebral Microbleeds, *WAIS-R* Wechsler Adult Intelligence Scale-Revised, *Assoc. Learning* Association Learning, *FAB* Frontal Assessment Battery, *SDS* Self-rating Depression Scale

Values are mean (SD) or n (%). ^a^ *p*-value of paired t-test or McNemar test

### Cognitive functions

**Fig. 1:**
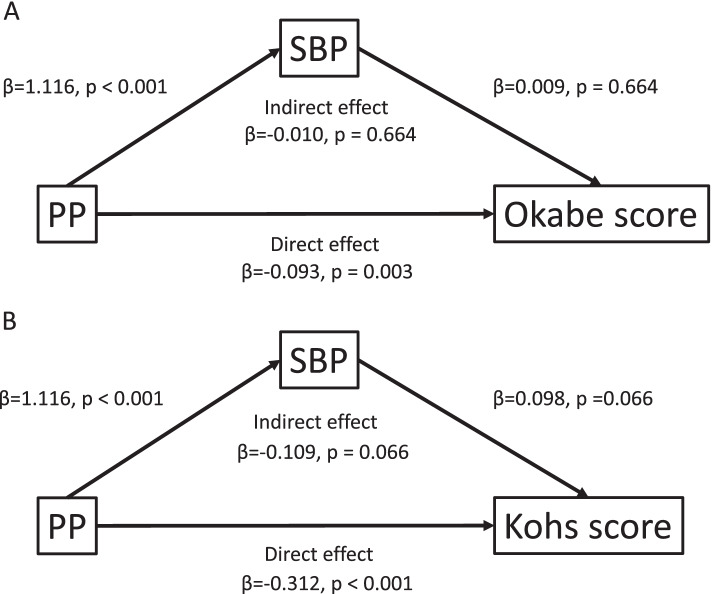
Mediation of the relationship between pulse pressure and cognitive function. **a** Measurement of systolic blood pressure mediation in the relationship between pulse pressure and Okabe’s test score. **b** Measurement of systolic blood pressure mediation in the relationship between pulse pressure and Kohs’ test score

The cognitive function scores for the two groups before and after propensity matching are presented in Supplemental Table and Table 2, respectively. Before matching, the high PP group showed lower cognitive function scores in Okabe’s test (*p* < 0.001), Kohs’ test (*p* < 0.001), FAB (*p* < 0.001), and SDS (*p* = 0.014) compared with the low PP group (Supplemental Table). After matching, the Okabe scores (especially mental control and digit span) and Kohs’ scores were still lower in the high PP group than in the low PP group (*p* < 0.05). Apathy scale scores did not show any difference between the groups before and after matching. To investigate whether the relationship between PP and cognitive function was affected by SBP, mediation analysis was performed. As shown in Fig. 1a the direct effect of PP on Okabe’s test was significant (effect size of -0.093), while the SBP mediated effect of PP on Okabe’s test was not significant. Similarly, as shown in Fig. 1b, the direct effect of PP on Kohs’ test was significant (effect size of -0.312), while the SBP mediated effect of PP on Kohs’ test was not significant. Thus, PP was significantly associated with Okabe’s and Kohs’ tests, but was not mediated by SBP (Table 3).

**Table 3:** The relationship between pulse pressure and cognitive function tested by mediation analysis

					95% Confidence Interval	
	Estimate	Std. Error	Z value	*P* value	Lower	Upper
Okabe						
Direct effects (PP → Okabe)	-0.093	0.031	-2.983	0.003	-0.153	-0.032
Indirect effects (PP → SBP → Okabe)	0.010	0.024	0.435	0.664	-0.037	0.058
Total effects (PP → Okabe)	-0.082	0.02	-4.181	< 0.001	-0.121	-0.044
Kohs						
Direct effects (PP → Kohs)	-0.312	0.077	-4.072	< 0.001	-0.462	-0.162
Indirect effects (PP → SBP → Kohs)	0.109	0.059	1.839	0.066	-0.007	0.226
Total effects (PP → Kohs)	-0.203	0.049	-4.172	< 0.001	-0.298	-0.107

*Abbreviation: PP* Pulse Pressure, *SBP* Systolic Blood Pressure

### Additional multiple linear regression analysis

The relationship between the cognitive function scores and PP was also examined by multiple linear regression analysis using the whole study sample. The detailed results are presented in Table 4. The effect of PP on Okabe’s test and Kohs’ test was significant, whereas the effect of SBP was not.

**Table 4:** Multiple linear regression analysis of cognitive function

Dependent variable	Independent variables				95% Confidence Interval		
		B	Std. Error	*P* value	Lower	Upper	VIF
Okabe	(constant)	58.506	1.427	< 0.001	55.708	61.305	
(R^2^ adjusted = 0.103)	Age	-0.195	0.014	< 0.001	-0.223	-0.167	1.194
	Sex	0.076	0.289	0.793	-0.490	0.642	1.117
	Hypertension	-0.336	0.317	0.290	-0.958	0.286	1.246
	Diabetes	-0.079	0.473	0.867	-1.006	0.848	1.046
	Hyperlipidemia	-0.014	0.275	0.960	-0.554	0.526	1.021
	Systolic BP	0.017	0.013	0.189	-0.008	0.043	2.711
	PP	-0.720	0.019	< 0.001	-0.109	-0.034	2.633
Kohs	(constant)	167.590	3.262	< 0.001	161.193	173.987	
(R^2^ adjusted = 0.284)	Age	-0.852	0.033	< 0.001	-0.917	-0.788	1.194
	Sex	-4.654	0.660	< 0.001	-5.948	-3.360	1.117
	Hypertension	-0.402	0.725	0.579	-1.824	1.019	1.246
	Diabetes	-0.560	1.081	0.604	-2.679	1.558	1.046
	Hyperlipidemia	-0.695	0.630	0.270	-1.930	0.539	1.021
	Systolic BP	-0.009	0.030	0.767	-0.067	0.049	2.711
	PP	-0.104	0.043	0.017	-0.189	-0.019	2.633

*Abbreviation: VIF* Variance Inflation Factor

## Discussion

In this study, we revealed that higher PP was associated with lower cognitive performance. Furthermore, our results indicate that these relationships were not mediated by SBP. However, we observed no significant relationship between PP and silent brain lesions.

To date, studies on the association between PP and cognitive function have inconsistent results, stemming mainly from the difference in age of the study populations. The subjects, who participated in our study, were individuals with an average age of 60 years; therefore, in this study, the impact of PP on cognitive performance of middle-aged populations wasfocused. Recently, consistent with our study, Zang et al. [6] in a study on 3009 subjects from the SPRINT-MIND revealed that higher PP was associated with poor cognitive performance. Similarly, Obisesan et al. [7], in a total of 3129 subjects from the Third National Health and Nutrition Examination Survey revealed that higher PP was associated with worse MMSE performance. Similar relationship between PP and cognitive function has also been demonstrated in longitudinal studies. Peters et al. [8], in a total of 3337 subjects from the HYVET cohort, revealed that higher PP was associated with an increased risk of dementia during a 2.2-year of follow-up. Similarly, other studies have revealed that PP predicts cognitive decline in community-dwelling individuals [9, 24]. On the other hand, studies in very old individuals oppose the findings of this study. Molander et al. [10] demonstrated that higher PP related to better cognitive function in a cohort of 476 participants aged 85 years or more. Similarly, Sabayan et al. [11] revealed that higher PP associates with lower annual declines in MMSE scores during the 3.2-year follow-up in the 572-participant cohort of the Leiden 85-plus Study. In the eldest, impaired vascular system function can result in low PP and hypoperfusion in the brain, which in turn may induce cognitive impairment. Further studies are needed to evaluate these age-dependent differences in the relationship between PP and cognitive function.

Interestingly, the relationship between PP and Okabe’s and Kohs’ tests was not mediated by SBP, even though SBP was strongly associated with PP and was previously linked with cognitive dysfunction [25, 26]. PP could be a surrogate marker of arterial stiffness [27] that represents the chronic effects of hypertension other than BP itself. Our results are consistent with previous reports showing that PP was a better predictor of cognitive impairment than BP [28, 29]. Thus, hypertension-associated changes in the brain might be detected by measures of arterial stiffness, such as PP, rather than SBP. Increased PP might represent diminished regulatory functions of vessels against pulsate blood flow, making the brain tissues more susceptible to direct injury.

In this study, PP was not associated with the prevalence of silent brain lesions, including SBI, PVH, SWML, and CMBs, indicating that PP might affect cognitive function independent of the burden of arteriosclerotic cerebral small vessel-related lesions. In previous reports, the relation between arterial stiffness the amount of Aβ deposition in the brain was suggested [30, 31]. A previous study revealed that arterial stiffness measured with peripheral pulse wave velocity significantly associates with the extent of Aβ deposition and the accumulation of Aβ in the brain over 2 years in elderly adults without dementia [30]. Since an increase in PP is recognized as arterial stiffness, it is speculated that increased PP causes decreased flow of brain interstitial fluid, thereby leading to decreased Aβ clearance along the perivascular space [32, 33] and accelerating the formation of Aβ plaques.

This study has several limitations. First, the cross-sectional study design limits causal inferences. More importantly, the application of the propensity score matching in this scenario (assuming the individual’s PP-level as an exposure) is not typical to the original concept of this analysis. Second, the subjects were recruited from a health examination cohort that might not properly represent the entire population of Japan. Third, the association between cognitive performance and brain lesions was not evaluated. Finally, we could not exclude the possibility of residual confounding by unmeasured determinants, such as medication, diet, or physical activity, which could have an effect on PP.

## Conclusion

In conclusion, our findings suggest that PP has a significant relationship with cognitive function among non-demented Japanese individuals. Higher PP was associated with lower general intelligence and visuospatial ability without SBP mediation. Future longitudinal studies are needed to explore the association between PP and cognitive decline in a representative sample of the Japanese population.

## Supplementary Information

**Additional file 1: Supplemental Table. **Physical, brain, and cognitive assessment in participants with lower and higher pulse pressure before matching.

## Data Availability

The raw data associated with this study are available from the corresponding author upon reasonable request.

## Abbreviations

MRI: Magnetic resonance imaging
PP: Pulse pressure
SBP: Systolic blood pressure
DBP: Diastolic blood pressure
BP: Blood pressure
CMB: Cerebral microbleed
PVH: Periventricular hyperintensity
WMH: White matter hyperintensity
SWML: Subcortical white matter hyperintensity
FAB: Frontal assessment battery
SDS: Self-rating Depression Scale
SBI: Silent brain infarct
