# Supplementary material for: Pulse pressure is associated with cognitive performance in Japanese non-demented population: a cross-sectional study
Source: BMC Neurol. 2022 Apr 11;22:137. doi: 10.1186/s12883-022-02666-6 (PMC8996505; doi:10.1186/s12883-022-02666-6)
Supplement: Supplementary file 1 — Additional file 1: Supplemental Table. Physical, brain, and cognitive assessment in participants with lower and higher pulse pressure before matching. [file 12883_2022_2666_MOESM1_ESM.pdf]

Supplemental table.

Physical, brain, and cognitive assessment in participants with lower and higher pulse pressure before matching

	Pulse pressure (mmHg)		
	Lower (<65)	Higher (≧65)	p value[a]
Systolic BP (mmHg)	124.3(14.3)	149.9(14.2)	<0.001
Diastolic BP (mmHg)	72.8(11.2)	75.8(11.5)	<0.001
PP (mmHg)	51.5(8.0)	74.1(7.3)	<0.001
SBI	111 (5.3)	45 (10.3)	<0.001
PVH grade≧3	119 (5.6)	51 (11.6)	<0.001
SWML	398 (18.9)	146 (33.3)	<0.001
CMBs	119 (5.6)	32 (7.3)	0.184
Okabe's test (shortened version of WAIS-R)	45.3 (7.1)	42.7 (7.5)	<0.001
Information	16.3 (2.7)	15.9 (2.8)	0.005
Mental control	12.2 (3.8)	11.0 (4.1)	<0.001
Digit span	9.1 (1.3)	8.8 (1.5)	<0.001
Assoc. learning	7.6 (3.1)	7.0 (3.1)	<0.001

Kohs' test	103.0 (18.3)	94.7 (18.1)	<0.001
FAB	16.1 (1.5)	15.6 (1.5)	<0.001
SDS	34.8 (7.5)	33.8 (7.7)	0.014
Apathy scale	11.2 (5.7)	11.2 (5.7)	0.995

Abbreviation: BP, blood pressure; SBI, silent brain infarction; PVH, periventricular hyperintensity; SWML, subcortical white matter hyperintensity; CMBs, cerebral microbleeds; WAIS-R, Wechsler Adult Intelligence Scale-Revised; Assoc. Learning, Association learning; FAB, frontal assessment battery; SDS, self-rating depression scale. Values are mean (SD) or n (%). a p-value of students' t-test or χ^2 test
